# Associations between alcohol use and outcome of psychological treatment in specialist psychiatric care – a cohort study

**DOI:** 10.3389/fpsyg.2024.1374339

**Published:** 2024-06-27

**Authors:** Jan Erik Lundkvist, Katarina Georgsson, Per Carlbring, Robert Johansson, Tomas Ljungberg, Sara Wallhed Finn, Therese Anderbro

**Affiliations:** ^1^Psychiatric Clinics, Region Sörmland, Nykoping, Sweden; ^2^Centre for Clinical Research Sörmland, Uppsala University, Uppsala, Sweden; ^3^Department of Psychology, University of Stockholm, Stockholm, Sweden; ^4^Karolinska Institute, Stockholm, Sweden

**Keywords:** psychological treatment, psychotherapy, hazardous use of alcohol, risky use of alcohol, harmful use of alcohol, alcohol dependence, outpatients, outcomes

## Abstract

**Background:**

Alcohol-related issues are widespread worldwide and are fairly substantial. Numerous studies have identified and clarified the effects and prevalence of alcohol use across different contexts. However, when it comes to the prevalence of alcohol in psychiatry and its impact on treatment outcomes compared to other patient groups, studies are relatively scarce, and results often vary, sometimes with different outcomes. This study focuses on investigating the effectiveness of psychological treatment in psychiatric clinics for outpatients, considering those with and without hazardous alcohol use under naturalistic conditions.

**Methods:**

Patients were recruited between 2012 and 2016 from psychiatric clinics in Sormland, Sweden, as part of the regular services. Patients completed symptom assessment instruments regarding depression, anxiety, quality-of-life, and alcohol consumption at the beginning of their psychological treatment, upon completion, and during a follow-up 1 year after completion. Completion of questionnaires was ongoing for some patients until 2021. A total of 324 patients were included in the study, distributed among 59 participating therapists.

**Results:**

Among all patients in the study, 30.2% showed hazardous alcohol use at the start of their psychological treatment, with a higher proportion being men. There was a significant reduction in the proportion of patients with hazardous use and a notable decrease in the mean audit score upon completion of psychological treatment. At follow-up, there was no significant change compared to completion. There were 31.2% of the patients who achieved recovery or improvement in the audit score upon completion of treatment. Patients with hazardous alcohol use consistently scored higher mean values on the symptom assessment instruments and lower on the quality-of-life instrument at the beginning. More individuals with hazardous alcohol use typically achieved better results across all outcome instruments at both at completion and follow-up.

**Conclusion:**

Patients with hazardous alcohol use demonstrate significant improvements in their alcohol consumption through standard psychological treatment in psychiatry, despite the treatment not specifically focusing on alcohol consumption. The progress/improvement appears to be largely maintained at follow-up. Moreover, patients with hazardous alcohol use tend to show greater progress across all outcome instruments. No significant gender differences were detected in this context.

## 1 Introduction

Studies have shown that psychotherapy can be effective for a wide range of psychiatric diagnoses (Lambert, 2013; Lazar, 2014; Munder et al., 2019), and psychological treatments have shown favorable outcomes for psychiatric outpatients within clinical settings (Nordmo et al., 2020; Lundkvist et al., 2023).

Alcohol-related issues are pervasive worldwide, where the prevalence of alcohol use disorders (AUDs) estimated at 8.5%, and a lifetime prevalence estimated at 20% (Slade et al., 2016). The prevalence of AUDs is significantly higher for men than for women (Glantz et al., 2020). The World Health Organization (WHO) defines different patterns of excessive alcohol use, classifying them as hazardous or harmful (Babor et al., 2001). The highest incidence of harmful alcohol consumption appears to be in Europe (Peacock et al., 2018).

Studies show that hazardous and harmful alcohol use is particularly prevalent among individuals with psychiatric disorders (Grant et al., 2015; Nehlin et al., 2017) and hazardous alcohol consumption is more frequent (Toftdahl et al., 2016). Nevertheless, there are a few studies that clarify alcohol consumption levels among patients in outpatient psychiatric care. In a previous Swedish study, hazardous or harmful alcohol consumption was found in approximately 22% of the women and 30% of the men (Eberhard et al., 2015). In a Danish study, an average of 25% of all patients with a psychiatric diagnosis had AUD (Toftdahl et al., 2016). At the same time, AUDs are generally both under-diagnosed and under-treated (van Amsterdam et al., 2022).

A Swedish Randomized Controlled Trial revealed that alcohol consumption patterns did not change for patients with depression who received targeted treatment for depression (Strid et al., 2019). Although some reduction in Alcohol Use Disorders Identification Test (AUDIT) scores was observed, the patients still maintained hazardous drinking levels according to the cut-off score. Hazardous alcohol use has been found to delay recovery among psychiatric outpatients (Bahorik et al., 2016). Women seem to seek and receive help to a higher extent than men for psychiatric problems (Lundkvist et al., 2023) they may also do so for alcohol-related problems (Livingston et al., 2021). There are studies that seem to show that women’s drinking decreases less than men after offered therapy (Livingston et al., 2021) but there is still uncertainty about the effect of different treatments depending on gender (Heinemans et al., 2014).

According to the Swedish guidelines, both harmful alcohol use and psychiatric problems should be treated simultaneously (Socialstyrelsen, 2019). In practice, harmful alcohol use and dependence are typically addressed by “substance use services,” while psychiatric symptoms are managed through psychiatric outpatient care. A recent study within psychiatric outpatient care found relatively few patients diagnosed with a substance use disorder (Lundkvist et al., 2023).

A common request from clinical practice, according to the authors’ experience, is that the alcohol use should be at a manageable level - to facilitate good engagement in the sessions and encourage patients to face anxiety instead of avoiding it by using alcohol - in order to benefit from psychological treatment in psychiatric outpatient care. If not, the harmful use or dependence is first treated through substance abuse services, according to clinical practice. In general, it seems that addiction treatment actions are only taken when alcohol problems are severe, despite suggestions that starting treatment earlier could be beneficial (Rehm et al., 2015). There are difficulties in assessing the actual treatment outcomes in patients with comorbid substance use disorder and depression/anxiety, as existing studies provide limited clarity (Hesse, 2009; Hides et al., 2019). The outcome of interventions targeting individuals with hazardous alcohol use and mental health problems is also ambiguous (Boniface et al., 2018). Further research in this field would be highly beneficial.

The aim of this study was therefore to highlight the following questions for patients undergoing outpatient psychiatric care:

- What is the extent of hazardous/harmful alcohol use at the start of psychological treatment, at completion and during follow-up?
- Do patients with hazardous/harmful alcohol use at the start experience any changes in their alcohol consumption levels by the time of treatment completion and follow-up?
- Are there differences in the outcome of psychiatric symptoms and quality-of-life (QoL) after completion of psychological treatment and at follow-up, between patients with hazardous/harmful alcohol use compared and those with a low risk of alcohol use at the outset?
- Are there gender differences in the above questions?

## 2 Materials and methods

### 2.1 Procedures

Patients were recruited from psychiatric outpatient clinics in the county of Sörmland, Sweden between 2012 and 2016. Participation was voluntary, with patients being invited to join the study when they started their psychological treatment. They completed questionnaires at the start, upon completion and during a follow-up, 1 year after the completion of treatment. All questionnaires had to be completed and answered by the patients themselves. A research assistant handled the questionnaires in accordance with the requirements of confidentiality and in line with the ethical statement. The last patient who submitted a completed questionnaire to be included in the study was in 2021. The patients were offered regular psychological treatment at a psychiatric clinic, focusing mainly on symptoms of anxiety and depression, possible personality syndromes, and neuropsychiatric conditions. Psychological treatment consistent with established clinical routines at the psychiatric clinic was used throughout this study, as described in detail previously (Lundkvist et al., 2023). An overall majority of therapists had basic or specialist training in psychotherapy (82%). Of these, most therapists had a cognitive behavioral or psychodynamic focus. For more detailed information see Lundkvist et al. (2023). The treatment did not focus specifically on alcohol consumption.

### 2.2 Sample description

#### 2.2.1 Participating patients

There were 324 patients who answered questionnaires at the start of their psychological treatment, specifying their gender as male or female, having participated in at least five sessions of psychological treatment to be included in the study. Diagnosis was made according to the current routines in outpatient care.

Among the 98 patients displaying hazardous use/harmful use/dependence on alcohol, 78 had hazardous use, 15 patients had harmful use, and five showed signs of dependence. In this study, therefore, hazardous, harmful, and dependence on alcohol have all been merged into the category of hazardous alcohol use. This was because it was not meaningful to make calculations based on the limited number of patients in the harmful and dependence categories at the time of assessment. Table 1 presents a summary of the demographic and clinical data pertaining to the participating patients, distributed by proportion of patients with no hazardous use and with hazardous alcohol use, based on the AUDIT scores at the start of the psychological treatment. No clinically relevant significant differences were observed between patients with and without hazardous alcohol use. Although there were significant differences in diagnoses, there were only marginal differences in the two main diagnoses: depression and anxiety. See Table 1. There is no significant association between diagnosis and AUDIT score either at the start of psychological treatment (0.416, tested using Kruskal–Wallis) or at completion (0.253), excluding the diagnosis “Alcohol and opiod dependence F10.2 and F11.2.”

**Table 1:** Characteristics between patients with and without hazardous/harmful alcohol use at the start of psychological treatment.

		**No hazardous use**	**Hazardous use of alcohol**	**All**	
	*N*	226	98	24	
**Age**	324	35.7 (12.2)	33.4 (11.9)	35.0 (12.2)	0.085^1^
**Gender**	324				0.094^2^
Woman		164 (72.6%)	62 (63.3%)	226 (69.8%)	
Man		62 (27.4%)	36 (36.7%)	98 (30.2%)	
**Marital status**	321				0.13^2^
Married or co-habiting		117 (52.0%)	41 (42.7%)	158 (49.2%)	
Single		108 (48.0%)	55 (57.3%)	163 (50.8%)	
**Children**	319				0.13^2^
No		94 (42.3%)	50 (51.5%)	144 (45.1%)	
Yes		128 (57.7%)	47 (48.5%)	175 (54.9%)	
**Highest level of education**	300				0.077^2^
Elementary school		35 (16.7%)	21 (22.1%)	56 (18.7%)	
Upper secondary		103 (49.0%)	49 (51.6%)	152 (50.7%)	
College/University		72 (34.3%)	20 (21.7%)	92 (30.6%)	
**Main diagnosis**	286				0.028^2^
Depression F32-34.*		47 (23.7%)	18 (20.5%)	65 (22.7%)	
Anxiety F40-44.*		90 (45.5%)	40 (45.5%)	130 (45.5%)	
Bipolar F30-31.*		14 (7.1%)	3 (3.4%)	17 (5.9%)	
Personality disorders F60.*		15 (7.6%)	10 (11.4%)	25 (8.7%)	
Hyperactivity disorders F90.*		26 (13.1%)	11 (12.5%)	37 (12.9%)	
Alcohol and opioid dependence F10.2.* and F11.2.*		0 (0.0%)	5 (5.7%)	5 (1.7%)	
Psychotic states F20.* and F23.*		2 (1.0%)	1 (1.1%)	3 (1.0%)	
Other		4 (2.0%)	0 (0.0%)	4 (1.4%)	
**Prior psychological treatment**	314				0.86^2^
No prior psychological treatment		37 (16.9%)	14 (14.7%)	51 (16.2%)	
Therapy shorter than one year		80 (36.5%)	37 (38.9%)	117 (37.3%)	
Therapy longer than one year		102 (46.6%)	44 (46.4%)	146 (46.5%)	
**Previous admission to hospital or treatment home due to mental illness**	309				0.60^2^
Not previously hospitalized		143 (65.8%)	56 (60.9%)	199 (64.4%)	
On one occasion		37 (17.1%)	20 (21.7%)	57 (18.4%)	
On several occasions		37 (17.1%)	16 (17.4%)	53 (17.2%)	
**Ongoing pharmacological treatment**	321				0.079^2^
No medication		49 (21.9%)	22 (22.7%)	71 (22.1%)	
Medicine in one category		113 (50%)	37 (37.8%)	150 (46.3%)	
Medicine in two or more categories		62 (27.4%)	38 (38.8%)	100 (30.9%)	
Current regular use of medications for mental disorders**	351				
Anxiolytics/sedatives/hypnotics		82 (36.6%)	43 (44.3%)	125 (38.9%)	0.19^1^
Antidepressant/mood stabilizer		140 (62.5%)	63 (64.9%)	203 (63.2%)	0.68^1^
ADHD*** medication		15 (6.7%)	6 (6.2%)	21 (6.5%)	0.87^1^
Analgetics		0 (0%)	2 (2.1%)	2 (0.6%)	0.031^1^

Data were obtained at pre-treatment. Tests used: ^1^Wilcoxon test; ^2^Pearson’s X^2^ test. * F32-35.* etc. diagnosis codes in International Classification of Diseases 10th Revision (ICD-10). ** A patient may use several different types of medications. *** ADHD: Attention deficit/hyperactivity disorder.

#### 2.2.2 Attrition and adherence

Based on the statistical summaries, there is no indication that the non-response of completed questionnaires was selective or that it exhibited any systematic differences compared to those who answered the questionnaires on several occasions. Therefore, it is unlikely that this non-response affected the outcomes in any decisive way; see further detail in 4.1 Dropouts and Methodological issues.

#### 2.2.3 Participating therapists

A total of 59 therapists agreed to participate in the study, each having patients who were included in the study. The majority of the therapists had between 1 and 7 patients (81%), and the remainder had between 9 and 21 patients participating in the study. The duration of psychological treatment provided by the therapist varied from 1 to 50 months, with a mean duration of 12.4 months. The number of sessions ranged from 5 to 140, with an average of 24.5 sessions.

### 2.3 Questionnaires

We chose the Alcohol Use Disorders Identification Test (AUDIT) as our instrument to measure the alcohol consumption levels, which is in line with the recommendations of the Socialstyrelsen, “The National Board of Health and Welfare” in Sweden (Socialstyrelsen, 2019). The AUDIT is a self-report instrument comprising 10 questions, focusing on the amount and frequency of alcohol consumption and its consequences (Saunders et al., 1993). It identifies hazardous and harmful alcohol use, but it is not a diagnostic tool (Bergman and Kallmen, 2002). As a screening instrument, AUDIT is easy to use (Socialstyrelsen, 2019), works in various settings, such as psychiatric clinics, and is suitable for monitoring changes in an individual’s drinking habits over time (Babor et al., 2001).

AUDIT offers different interpretation models and cutoffs for alcohol consumption levels. We chose a threshold of six points for hazardous alcohol use for women and eight points for men (Bergman and Kallmen, 2002). This distinction takes into consideration that women tend to reach higher blood-alcohol levels than men with the same alcohol intake, and the risks of medical alcohol-related harm are higher for women (Bradley et al., 1998). In addition, the following cutoffs have been used for harmful use (≥16) and for dependence (≥20) (Babor et al., 2001).

Other outcome instruments: Beck Anxiety Inventory (BAI) – was used to measure anxiety (Beck et al., 1988); Montgomery Åsberg Depression Rating Scale Self-Rated (MADRS-S) – was used to measure depression (Montgomery and Asberg, 1979); Symptom Check List −90 (SCL-90) - was used to describe the overall presence of psychiatric symptoms and their severity, (Derogatis et al., 1973) and Quality of Life Enjoyment and Satisfaction Questionnaire – Short Form (Q-LES-Q-SF) (Endicott et al., 1993) – was used to measure quality-of-life.

### 2.4 Data management

The results are based on an analysis of completed questionnaires. Instances of missing questionnaires were largely due to challenges faced by therapists, such as forgetting to distribute questionnaires upon completion of therapy. Difficulties in obtaining questionnaires arose, for example, when patients completed the treatment via telephone instead of at the clinic, where therapists could directly hand out questionnaires to them. However, there is no statistically significant difference between patients who completed the questionnaires once or on multiple occasions regarding diagnosis, medication, age, gender, ratings on symptom assessment instruments and quality of life instruments (Lundkvist et al., 2023). See also 4.1 Dropouts and Methodological issues.

### 2.5 Statistical analyses

#### 2.5.1 Between-group differences

Wilcoxon’s test was used for continuous data when comparing between groups, while T-test was used for paired comparisons. Pearson’s X^2^-test was used to test for differences in frequencies between groups, while McNemar’s test was used to test for differences in frequencies for paired comparisons.

#### 2.5.2 Reliable change and clinically significant change

Reliable change (RC) calculates if a change on a psychological test is both reliable and statistically significant (Jacobson and Truax, 1991). RC reports whether the individual patient’s outcome is a positive change, no change, or deterioration. Clinically significant change (CSC) refers to the change from a typical dysfunctional or problematic score to one corresponding to a ‘healthy’ population (Jacobson and Truax, 1991). There are three ways to calculate if the changes also meet the conditions for CSC. In the current study, we chose method A, as it was considered an appropriate way to determine a real change in the selected outcome instruments. Additionally, there were no available studies showing normal population values for MADRS-S. First, the patient must fulfill the conditions for RC. Second, the patient has to move more than 2 SD from the mean of the “problem group.”

When calculating RC and CSC for BAI, Madrs-S, SCL-90, and Q-Les-Q, cutoff values were used to exclude patients who, at the start of the psychological treatment, could be considered as “healthy” based on their self-assessment instrument scores. The selected cutoff values were BAI ≤ 10 (Westbrook and Kirk, 2005), SCL-90 ≤ 66 = 0.73/item (Carrozzino et al., 2016), Q-LES-Q ≥ 60 = 82% (Schechter et al., 2007), and MADRS-S ≤ 13. The MADRS-S cutoff was selected based on scale comparisons with BAI, matching the level of difficulty.

For further background information about procedures, recruitment, inclusion and exclusion criteria, administration of questionnaires, participating patients, attrition rates and adherence, participating therapists, data management, and statistical analyses, see Lundkvist et al., 2023.

## 3 Results

### 3.1 Extent of hazardous alcohol consumption

Approximately one-third of the patients exhibited hazardous alcohol use at the start of their psychological treatment, see Table 2. The proportion of men with hazardous use was nearly 10% higher than that of women at the start. There was a significant reduction in the proportion of patients with hazardous use upon completion for all patients together, especially among men. No significant difference was found between completion and follow-up.

**Table 2:** Number of patients (%) with and without hazardous alcohol use at the start, completion, and follow-up of psychological treatment; for all, female and male.

	**Start** N 324	**Completion** N 203	**Follow-up** N 138	***P*-value**^**1**^ Sta-Com*	**P-value**^**1**^ Com-FU**
**All**					
No hazardous use	226 (69.8%)	165 (81.3%)	107 (77.5%)	<0.001	0.752
Hazardous use	98 (30.2%)	38 (18.7%)	31 (22.5%)		
**Female**					
No hazardous use	164 (72.6%)	119 (78.8%)	77 (74.8%)	0.066	1.000
Hazardous use	62 (27.4%)	32 (21.2%)	26 (25.2%)		
**Male**					
No hazardous use	62 (63.3%)	46 (88.5%)	30 (85.7%)	0.001	0.480
Hazardous use	36 (36.7%)	6 (11.5%)	5 (14.3%)		

Paired comparisons. *Test used: ^1^McNemar.* * *Start-completion.* ** *Completion-Follow-Up.*

### 3.2 AUDIT score

A significant decrease was seen in the mean AUDIT score among patients with hazardous alcohol use, from the start and completion stages for women and men separately and all together, see Table 3. At the follow-up, scores generally showed a slight increase. There was no significant increase among patients with hazardous alcohol use between completion and follow-up. There was a significant increase in those without hazardous alcohol use between completion and follow-up for the entire group, but it does not appear to be divided by gender.

**Table 3:** AUDIT score at the start, completion, and follow-up for all, for female and male, for with and without hazardous alcohol use.

	**Start** M (Md) Q1-Q3*	**Completion** M (Md) *Q1-Q3*	**Follow-up** M (Md) Q1-Q3	***P*-value**^**1**^ Sta-Com**	***P*-value**^**1**^ Com-FU***
**All**	**N324**	**N203**	**N138**		
No hazardous use	2.2 (2.0) *0.2–3.0*	2.0 (1.1) *0.0–3.0*	2.9 (2.0) *0.0–4.0*	0.206	0.028
Hazardous	12.3 (11.0) *8.9–16.0*	7.9 (6.0) 4.4–12.0	8.3 (7.0) *3.5–11.0*	<0.001	0.286
**Female**	**N226**	**N151**	**N103**		
No hazardous use	1.9 (2.0) *0.8–3.0*	1.7 (1.0) *0.0–3.0*	2.5 (1.0) *0.0–3.0*	0.364	0.141
Hazardous	12.0 (11.0) *8.0–15.8*	8.3 (7.0) *5.0–12.0*	8.2 (7.5) *3.8–11.0*	<0.001	0.239
**Male**	**N98**	**N52**	**N35**		
No hazardous use	3.0 (3.0) *0.2–5.0*	3.0 (3.0) *1.0–4.6*	3.9 (4.0) *1.8–7.0*	0.272	0.100
Hazardous	12.9 (11.0) *9.0–16.0*	6.9 (6.0) *3.0–8.2*	8.6 (7.0) *4.5–13.0*	0.005	0.780

Paired comparisons. Test used: ^1^Paired *T*-test. * *Mean value (Median value) First and third quartiles.* ** *Start-completion.* *** *Completion-Follow-Up.*

In a supplementary analyses, using One-Way-ANOVA, a comparison was also made between the genders’ change in AUDIT score at the time of measurement. No significant differences were detected for either no hazardous use (0.739) or hazardous use of alcohol (0.379) between start and completion. No significant differences were detected for either no hazardous use (0.423) or hazardous use of alcohol (0.520) between completion and follow-up.

### 3.3 Proportion of improved, unchanged and deteriorated patients with hazardous use of alcohol at the start in terms of AUDIT score – measured at completion and follow-up

Almost one-third, 31.2%, attained a recovered or improved status on the AUDIT score upon completion of psychological treatment, see Table 4. At follow-up, this proportion had increased to 42.9%, with the change consisting of fewer individuals classified as recovered and more classified as improved compared to the completion stage. The percentage of unchanged cases decreased from 65.6% upon completion to 48.6% at time of follow-up, while for deteriorated cases, it increased from 3.3% at completion to 8.6% at the follow-up assessment.

**Table 4:** RCI and CSC.

**Completion**						**Follow-up**				
	*N*	Recovered	Improved	Unchanged	Deteriorated	*N*	Recovered	Improved	Unchanged	Deteriorated
		*N* (%)	*N* (%)	N (%)	*N* (%)		*N* (%)	*N* (%)	*N* (%)	*N* (%)
**Hazardous**	61	9 (14.8%)	10 (16.4%)	40 (65.6%)	2 (3.3%)	35	8 (22.9%)	7 (20.0%)	17 (48.6%)	3 (8.6%)

Outcome for patients who estimated hazardous alcohol use at the start. Number and percentage of patients who recovered (i.e., achieved reliable change and clinically significant change), improved (reliable positive change), remained unchanged, or deteriorated (reliable negative change) on the AUDIT score upon completion of therapy and at follow-up.

### 3.4 Outcome for psychiatric symptoms and quality-of-life at completion and follow-up based on alcohol grouping at the start

For women and men all together, higher mean scores for patients with hazardous alcohol use on all symptom assessment instruments and lower mean scores on the quality-of-life instrument were found at the start of psychological treatment, see Table 5. There were significant differences observed on the BAI and SCL-90 instruments. Upon completion of treatment, there were no significant differences in any of the outcome instruments between the level of alcohol use when the groups were kept intact with those at the start. Similarly, no significant differences were found in any of the outcome instruments at follow-up among the same groupings as at the start.

**Table 5:** Mean values (SD) at the start, completion, and follow-up of psychological treatment on the outcome instruments for all patients and categorized by gender, distinguishing between patients with and without hazardous alcohol use. The patients who rated themselves as non-hazardous alcohol users or hazardous users at the start belong to the same groupings at completion and follow-up, regardless of their alcohol consumption at those latter points in Table 5.

	**Start**				**Completion**				**Follow-up**				*P*-value^1^
	*N*	No hazardous use *N* 226	Hazardous use *N* 98	*P*-value^1^	N	No hazardous use at start** N 145	Hazardous use at start ** N 64	*P*-value^1^	*N*	No hazardous use at start** N 104	Hazardous use at start** N 36
**All**												
BAI	324	23.4 (11.8)	27.2 (12.3)	0.014*	209	18.2 (12.1)	17.2 (11.5)	0.61	140	17.8 (13.1)	20.2 (13.6)	0.36
MADRS-S	324	23.2 (9.1)	25.4 (8.3)	0.069	206	16.8 (10.6)	16.1 (9.2)	0.84	140	16.2 (10.6)	17.5 (10.3)	0.42
SCL-90	324	131.3 (57.5)	160.9 (49.1)	<0.001*	207	102.8 (67.4)	101.2 (62.8)	0.96	140	103.2 (67.3)	111.6 (67.9)	0.41
Q-LES-Q	324	37.6 (9.4)	36.6 (8.4)	0.42	209	42.4 (11.2)	44.2 (10.8)	0.36	140	42.3 (11.3)	42.0 (12.3)	0.62
		N 164	N 62			*N 112*	*N 43*			*N 76*	*N 29*	
**Female**												
BAI	226	25.1 (11.5)	29.9 (12.2)	0.015*	155	20.1 (12.1)	18.9 (11.7)	0.66	105	19.9 (13.4)	21.5 (14.5)	0.71
MADRS-S	226	23.9 (9.2)	25.3 (7.9)	0.45	153	17.7 (10.6)	16.4 (9.5)	0.61	105	16.9 (11.0)	18.7 (10.4)	0.32
SCL-90	226	138.7 (54.3)	168.2 (47.3)	< 0.001*	155	112.9 (66.6)	107.7 (63.5)	0.67	105	111.5 (68.8)	118.9 (72.1)	0.50
Q-LES-Q	226	36.8 (9.3)	35.4 (8.9)	0.34	155	41.2 (11.1)	44.1 (11.2)	0.18	105	41.2 (11.5)	41.8 (12.6)	0.83
		N 62	N 36			*N 33*	*N 21*			*N 28*	*N 7*	
**Male**												
BAI	98	18.8 (11.4)	22.7 (11.2)	0.11	54	11.9 (9.8)	13.7 (10.5)	0.44	35	12.2 (10.5)	14.9 (7.0)	0.31
MADRS-S	98	21.5 (8.9)	25.6 (9.2)	0.032*	53	13.6 (10.3)	15.5 (8.7)	0.31	35	14.4 (9.5)	12.4 (9.1)	0.63
SCL-90	97	111.5 (61.6)	148.3 (50.2)	0.002*	52	68.8 (59.2)	86.7 (60.5)	0.21	35	80.8 (58.6)	81.5 (36.9)	0.70
Q-LES-Q	98	39.7 (9.5)	38.5 (7.3)	0.48	54	46.6 (10.9)	44.4 (10.3)	0.48	35	45.3 (10.3)	42.7 (12.0)	0.82

^1^ Wilcoxon test. * Significant outcomes. ** Belong to the same cluster of patients as at the start of psychological treatment regardless their rated audit score at completion and at follow-up.

Disaggregation by gender revealed a higher mean score for both women and men with hazardous alcohol use on all symptom assessment instruments and lower mean scores on the quality-of-life instrument at the start, see Table 5. Significant differences were found for both women and men on the SCL-90. There was a significant difference on the BAI for women and a significant difference for men on Madrs-S. At treatment completion, there were no significant differences in any of the outcome instruments, based on gender, between patients with and without hazardous alcohol use. Similarly, at follow-up, there were no significant differences in any of the outcome instruments observed for either women or men.

### 3.5 Outcome on RC and CSC between start and completion

Between 42.1 and 54% of patients who reported no hazardous alcohol use at the beginning achieved improved or recovered outcomes upon completion, as indicated by the symptom assessment instruments, compared with between 63.2 and 79.7%, for patients with hazardous alcohol use, see Table 6. Concerning the quality-of-life instrument, 46.9% of patients with hazardous alcohol use showed improvements or recovery, compared to 33.3% of patients without hazardous use. The proportion unchanged is larger on all outcome instruments at completion for patients without hazardous alcohol use, ranging between 31.7 and 61.1%, compared to the hazardous users, which ranged between 11.9 and 43.8%.

**Table 6:** RC and CSC.

		**Completion**					**Follow-up**			
	*N*	Recovered	Improved	Unchanged	Deteriorated	*N*	Recovered	Improved	Unchanged	Deteriorated
		*N* (%)	*N* (%)	*N* (%)	*N* (%)		*N* (%)	*N* (%)	*N* (%)	*N* (%)
**BAI**										
No haz	121	19 (15.7%)	32 (26.4%)	60 (49.6%)	10 (8.3%)	84	10 (11.9%)	29 (34.5%)	34 (40.5%)	11 (13.1%)
Haz	57	13 (22.8%)	23 (40.4%)	18 (31.6%)	3 (5.3%)	33	5 (15.2%)	12 (36.4%)	14 (42.4%)	2 (6.1%)
**Madrs-S**										
No haz	118	40 (33.9%)	17 (14.4%)	52 (44.1%)	9 (7.6%)	84	26 (31.0%)	17 (20.2%)	36 (42.9%)	5 (6.0%)
Haz	59	19 (32.2%)	19 (32.2%)	19 (32.2%)	2 (3.4%)	34	9 (26.5%)	3 (8.8%)	20 (58.8%)	2 (5.9%)
**SCL-90**										
No haz	126	31 (24.6%)	37 (29.4%)	40 (31.7%)	18 (14.3%)	86	13 (15.1%)	33 (38.4%)	18 (20.9%)	22 (25.6%)
Haz	59	22 (37.3%)	25 (42.4%)	7 (11.9%)	5 (8.5%)	35	10 (28.6%)	13 (37.1%)	6 (17.1%)	6 (17.1%)
**Q-Les-Q**										
No haz	144	15 (10.4%)	33 (22.9%)	88 (61.1%)	8 (5.6%)	103	5 (4.9%)	18 (17.5%)	74 (71.8%)	6 (5.8%)
Haz	64	13 (20.3%)	17 (26.6%)	28 (43.8%)	6 (9.4%)	36	10 (27.8%)	5 (13.9%)	14 (38.9%)	7 (19.4%)

Results at completion and follow-up in relation to the starting values, for patients who scored at or above the cut-offs on the symptom assessment instruments and at or below the cut-off on the quality-of-life instrument, at the start of psychological treatment – divided between those with and without hazardous alcohol use. For applied cut offs see subsection 2.5.2. Number and percentage of patients who reached recovered status (i.e., achieved reliable change and clinically significant change), improved (reliable positive change), remained unchanged, or deteriorated (reliable negative change) on each outcome instrument at the completion of therapy and follow-up. BAI, Beck Anxiety Inventory; MADRS-S, Montgomery-Åsberg Depression Rating Scale - Self-report; SCL-90, Symptom Checklist-90; Q- LES-Q, Quality of Life Enjoyment and Satisfaction Questionnaire; AUDIT, Alcohol Use Disorders Identification Test.

### 3.6 Outcome on RC and CSC between start and follow-up

Between 46.4 and 53.5% of patients without hazardous alcohol use at the start achieved improved or recovered status at follow-up, based on the symptom assessment instruments, compared to between 35.3 and 65.7% for patients with hazardous alcohol use at the same time of measurement, see Table 6. Regarding the quality-of-life instrument, 41.7% of patients with hazardous alcohol use showed improvement or recovery, while 22.4% of patients without hazardous alcohol use reached the corresponding level. The proportion of those unchanged ranged from 20.9 to 42.9% on the symptom assessment instruments for patients without hazardous use at the start. The corresponding proportion for patients with hazardous alcohol use ranged from 17.1 to 58.8%. Concerning the quality-of-life instrument, 71.8% of patients without hazardous alcohol use remained unchanged compared with 38.9% for patients with hazardous alcohol use. The proportion of patients who deteriorated was higher on all symptom assessment instruments for those without hazardous alcohol use, ranging from 6.0 to 25.6%, compared to hazardous users, whose range was 5.9 to 17.1%. The opposite was observed on the quality-of-life instrument, where 19.4% of hazardous alcohol users deteriorated compared to 5.8% of the non-hazardous users.

### 3.7 Significance calculation of proportions

A significant difference was observed between patients without hazardous alcohol use and those with hazardous alcohol use on Madrs-S and SCL-90 at the completion of psychological treatment regarding the proportion of patients divided among RC, CSC, unchanged, and deteriorated categories, see Table 7. Moreover, a significant difference was found on the Q-Les-Q at follow-up.

**Table 7:** Significance calculations on the proportions of RC, CSC, unchanged, and deteriorated categories between patients without hazardous alcohol use and those with hazardous alcohol use for the respective symptom and quality-of-life instruments.

	***P*-value**	
	**Completion**	**Follow-up**
BAI	0.077	0.730
MADRS-S	0.034	0.344
SCL-90	0.009	0.344
Q-Les-Q	0.078	<0.001

Pearson’s X^2^.

## 4 Discussion

### 4.1 The extent of hazardous/harmful alcohol use

Even though a majority of patients did not report hazardous alcohol use, almost a third had hazardous/harmful alcohol use. These results are essentially in line with previous studies, thus confirming the approximate extent of hazardous/harmful alcohol use in psychiatric care (Eberhard et al., 2015; Toftdahl et al., 2016; van Amsterdam et al., 2022). Women showed a lower proportion of hazardous/harmful alcohol use, 27.4% compared to men, 36.7%, which also correlates with the findings in other studies (Eberhard et al., 2015; Glantz et al., 2020).

The proportion of patients with hazardous alcohol use decreased significantly from the start to the completion of treatment, indicating a positive impact of psychological treatment on alcohol consumption. When considering gender, the major difference is evident among men, while the difference for women was not statistically significant. One explanation may be the proportion of men with hazardous alcohol use at the start of treatment was clearly higher than that of women, providing more opportunities for improvement among men. At follow-up, there was a decrease in the proportion of non-hazardous alcohol users, but there was no significant increase in hazardous alcohol users. This indicates that patients largely maintained the positive results obtained upon completing their psychological treatment.

### 4.2 Level of alcohol consumption

A consistent and significant reduction in alcohol consumption among hazardous alcohol use patients was observed at the completion of the treatment. These reductions seemed largely maintained 1 year after completing psychological treatment. Women appeared to have achieved, on average, slightly better sustained outcomes at follow-up compared to men, but these differences were not statistically significant. It is notable that patients without hazardous alcohol use at the start showed a significant increase in their AUDIT score between completion and follow-up. The difference does not appear in calculations divided by gender, but in a combined calculation. This finding may be worth investigating further to try to explain and understand its implications.

Among patients with hazardous alcohol use at the start, approximately one-third, achieved recovery or improvement on the AUDIT scale by treatment completion. Given the significant increase in patients without hazardous alcohol use and the decrease in the average AUDIT score at completion, the percentage of patients who recovered or improved is approximately at expected level. At follow-up, the proportion of those who had recovered or improved increased, leading to a substantial reduction in the unchanged category. Nonetheless, the findings at follow-up are somewhat questionable, as previous outcomes have indicated a decline, to some extent, during this period. One explanation could be the relatively small number of patients in the follow-up group, so the results of individual patients may have a larger impact. The majority of patients with hazardous alcohol use at the start showed no clinically significant improvement in their AUDIT score by treatment completion. This observation, to some extent, balances the previous findings in the study.

### 4.3 Outcome of psychiatric symptoms and quality-of-life

When comparing patients with hazardous alcohol use at the beginning to those without, it appears that the former group consistently exhibits higher average values in symptom assessment and slightly lower quality-of-life scores. There are significant differences among the alcohol consumption groups in some of the symptom assessment instruments. However, by the time of treatment completion, there were no longer any significant differences between the alcohol use groups, and the mean scores of both comparative groups become quite similar, indicating that those with hazardous alcohol use at the start have, on average, improved their performance more than those without hazardous use on the symptom assessment instruments. The differences in means at completion may be explained by the fact that hazardous users are more likely to show progress, as they have consistently higher mean scores on the symptom assessment instruments at the start. Nevertheless, it is doubtful that these differences can be explained solely by regression to the mean, given that waiting times for psychological treatment from the time of application to psychiatry and being offered psychological treatment are usually several months, sometimes up to a year and even longer for some patients. Furthermore, a significant majority of patients have already started their medical treatment before the psychological treatment begins. In addition, it can be concluded that improvements tend to cease at the completion of psychological therapy and, to some extent, deteriorate 1 year after completion of treatment.

For the quality-of-life instrument, there are marginal differences between the groups. Even at follow-up, the groups remain relatively intact among themselves in terms of results. There is a slight tendency for hazardous users to experience slightly more decline in the symptom assessment items at follow-up, but no significant differences are observed between the patient groups that initially had non-hazardous use and hazardous use.

In terms of scores on the outcome instruments at start, completion and follow-up, the relationship between patients with and without hazardous alcohol use is similar for women and men. However, women have consistently, with only one exception, higher average scores at all measurement points on the symptom assessment instruments and slightly lower on the quality-of-life instruments than men, which may indicate that women are somewhat more psychiatrically burdened.

When comparing patients without hazardous alcohol use to those with hazardous use regarding the number/proportion of patients who achieved recovery or improvement between the start and completion, it is evident that the latter group generally has a better outcome across all outcome instruments. Patients without hazardous alcohol use show a larger proportion that is unchanged across all outcome instruments at completion. Furthermore, the proportion of deterioration is higher for patients without hazardous alcohol use on all symptom assessment instruments, whereas the reverse applies for the quality-of-life instrument, i.e., indicating lower outcomes for those with hazardous alcohol use. The largest statistical difference between the alcohol consumption groups in terms of proportions at completion was observed on the Madrs-S and SCL-90 scales.

Similarly, when comparing patients without hazardous alcohol use to those with hazardous use regarding the number/proportion of patients who were recovered or improved between the start and follow-up, the latter group still shows better outcomes on SCL-90, BAI, and Q-Les-Q, while the reverse is true for Madrs-S. The results indicate that patients with hazardous alcohol use who have a distinct score on the Madrs-S find it more difficult to maintain a sustained score from completion to follow-up compared to patients without hazardous use and compared to scores on other outcome instruments. It would be interesting to investigate whether patients with hazardous alcohol use and a distinct depression problem also had more difficulty achieving improvement in alcohol consumption levels, in line with the Strid et al. (2019) study. A statistically significant difference exists between the alcohol consumption groups in terms of QoL. A considerably larger proportion of hazardous users show improvement, but at the same time, a significant proportion of hazardous users experience deterioration at follow-up, which is an interesting observation. It appears that the quality-of-life outcome at follow-up, in comparison, either significantly improves or significantly deteriorates for patients who had hazardous alcohol use at the start.

### 4.4 Summary of outcomes by gender

There is some limited variation between men and women in terms of risky alcohol use during the period of psychological treatment. The proportion of men going from hazardous to non-hazardous alcohol use at completion is larger than for women. On the other hand, when the amount of alcohol is highlighted between start and completion, no differences can be detected. It is possible that women with hazardous alcohol use may be able to maintain the achieved reduction in alcohol somewhat better than men at follow-up, especially if we study mean values. The median values, however, show fairly similar outcomes. We can conclude that there are no significant changes related to gender in AUDIT scores between the measurement occasions. Overall, the study has not been able to detect any significant differences in risky alcohol use that can be explained by gender differences; the patterns appear to be similar regardless of gender.

### 4.5 Dropouts and methodological issues

Overall, there were few significant differences in background variables between non-hazardous users and hazardous/harmful users; see Table 1. The significant findings were of limited clinical value, mainly related to diagnosis and analgesic use. There were many diagnostic groups, which meant that a few patients could have a significant impact on the outcomes. In the major diagnostic groups, such as anxiety and depression, the proportions are evenly distributed, and no other remarkable differences can be detected on visual inspection. Regarding the significance of analgesic use, there are too few patients in the study receiving this medication to draw any meaningful conclusions. In summary, there is no indication in the results that any of the background factors examined could explain differences in outcomes between the groups.

There were no significant differences in the proportions of non-hazardous and hazardous alcohol users between patients who completed the questionnaires only on one occasion versus on several occasions, see Supplementary Table S1. There were no significant differences in the results of the outcome instruments at the start of psychological treatment for patients with hazardous/harmful alcohol use based on whether they were excluded from the study due to too few sessions or if they answered the questionnaires on one or more occasions, see Supplementary Table S2. The dropout rates for completed questionnaires and participating patients in the study can be considered random rather than systematic. There are no indications that the lack of completed questionnaires at completion or follow-up would explain the results. It is also worth noting that dropout in the study only pertains to patients who did not answer the questionnaires, which is not the same as not completing the initially agreed psychological treatment for various reasons. All patients, regardless of their reasons for discontinuing psychological treatment, were given the opportunity to complete questionnaires at both completion and follow-up.

Regarding the dropouts (individuals not answering the survey), the proportion of patients with hazardous/harmful alcohol use at the start decreased to the same extent as patients without hazardous use by completion, see Supplementary Table S3. At follow-up, there was also no significant difference between the groups. Patients with hazardous alcohol use seem as likely to complete psychological treatment as other patients (Hunt and Delgadillo, 2022) and they also seem to be at least as likely to answer questionnaires at completion and follow-up as other patients.

Perhaps the proportion of patients with hazardous use in relation to the number of patients with harmful alcohol use and dependence might have affected the study’s outcome, as the number of patients with hazardous use was in the clear majority, see Supplementary Table S4. We could assume that it is easier for patients with hazardous alcohol use to make positive changes and achieve non-hazardous use compared to those with more severe alcohol consumption.

The study did not choose to make any multiplicity adjustment, such as the Bonferroni correction, because its focus is not on a single significant value but on identifying patterns. There is an increased risk of type-2 error when using the Bonferroni correction, i.e., that we do not detect any significance at all, although there may be significant results. Bonferroni correction also assumes that the different tests are independent of each other, which is not the case in this study. In the current study, outcome instruments mostly show results in a comparable direction, which strengthens the credibility of the outcome.

The study has not chosen to impute values for non-response at completion and follow-up. The assessment has been that the gain would be relatively small. Admittedly, more data would be obtained, but on the other hand, the data would add extra uncertainty, which is why the choice was not made. The exception would have been if we had missing values in certain variables that we wanted to adjust for in a model.

### 4.6 Limitations

This study has several limitations that could have affected the results. Neither the therapists nor the patients were randomized to participate, and there was no control group. There was no control over which patients were not included in the study and on what basis these decisions were made by the patients or therapists. For example, in outpatient psychiatric care, patients might be treated for acute problems when they attend psychotherapy for the first time, which is why submission of questionnaires is not a priority. This increases the risk of questionnaires being forgotten, as well as the offers to participate in the study.

The outcome of the study is based on patients’ reported scores. Although the AUDIT is an established and well-used instrument that has been reliability tested and validated, the results might have been even more reliable if an objective test had also been used. However, it is unlikely that the study could have been carried out with all the extra work for therapists and patients, as well as the ethical considerations it entailed.

Attrition in the study may affect the outcome of the study. We do not know how or if the results would be affected if we had received completed questionnaires from those who did not respond at completion and/or at follow-up. The proportion of men who did not answer questionnaires at completion compared to women was slightly higher, we do not know how or if the results were affected by this non-response. However, as shown in Supplementary Table S3, there are no significant differences in non-response between patients who had or did not hazardous use of alcohol at start, nor significant differences between genders.

Moreover, we do not know to what extent specific medications or medication adjustments during psychological treatment or life events outside the treatment room affected the results. This was a real-life study where many factors could have affected the outcomes as the treatment took place in a realistic clinical environment. The fact that the study involves a mix of patients with different problems and various therapeutic approaches is a limitation that reduces internal validity. However, this increases the external validity.

## 5 Conclusion

At the completion of psychological treatment, there is a good chance that patients with hazardous alcohol use at the start have considerably reduced their alcohol consumption, based on:

- a significantly decreased percentage/number of hazardous users.
- a significantly decreased mean value among hazardous users on the AUDIT score.
- significant improvements on the AUDIT score for almost a third of the hazardous users.

At the same time, the majority of patients with hazardous use at the start did not achieve any statistical change at completion. Some of these patients might have made minor improvements that were not statistically significant but may have gone from being a hazardous alcohol user to a non-hazardous one. The result should also be seen from the perspective that patients were offered regular psychological treatment, without any focus on what was revealed by the AUDIT screening; nevertheless, a real reduction in alcohol consumption seems to have been observed. Despite these good improvements, this group of patients might still benefit from further support specifically regarding their alcohol consumption during the psychological treatment.

The improvements observed in AUDIT scores at completion seem to be maintained, to a fairly high extent, at follow-up.

Consistently across all measures, patients with hazardous alcohol use at baseline showed the most improvements. Based on these results, we can conclude that patients with hazardous alcohol use achieve at least as good a therapeutic outcome on the symptom assessment instruments as patients without hazardous use, as shown in other studies (Hunt and Delgadillo, 2022). This means that therapists, clinicians, and healthcare facilities need not hesitate to treat patients with hazardous alcohol use for anxiety and depression problems under the assumption that their alcohol consumption makes it difficult to benefit from psychologicaltreatment.

No substantial differences were detected between genders in terms of outcomes related to alcohol consumption or results on other symptom assessment instruments or QoL.

## Data availability statement

The original contributions presented in the study are included in the article/Supplementary material, further inquiries can be directed to the corresponding author/s.

## Ethics statement

The studies involving humans were approved by The Ethics Review Authority in Sweden approved the study, approval no 2012/1308-31 and after additions 2016/310-32. The studies were conducted in accordance with the local legislation and institutional requirements. Written informed consent for participation in this study was provided by the participants’ legal guardians/next of kin.

## Author contributions

JL: Supervision, Project administration, Methodology, Investigation, Formal analysis, Conceptualization, Writing – review & editing, Writing – original draft. KG: Writing – original draft, Project administration, Methodology, Investigation. PC: Writing – review & editing, Supervision, Methodology. RJ: Writing – review & editing, Supervision, Methodology. TL: Writing – review & editing, Supervision, Methodology. SW: Writing – review & editing, Supervision, Methodology. TA: Writing – review & editing, Supervision, Methodology, Investigation, Formal analysis.
