# Supplementary material for: Associations between alcohol use and outcome of psychological treatment in specialist psychiatric care – a cohort study
Source: Front Psychol. 2024 Jun 27;15:1374339. doi: 10.3389/fpsyg.2024.1374339 (PMC11238639; doi:10.3389/fpsyg.2024.1374339)
Supplement: Supplementary file 1 [file Data_Sheet_1.docx]

Supplementary Material

# Supplementary Data

**Table 1**  Proportion of patients with and without hazardous/harmful alcohol use at the start of their psychological treatment by category.

|  | **1*** | **2*** | **3*** | **4*** | **5*** | **All** | **P-value** |
| --- | --- | --- | --- | --- | --- | --- | --- |
|  | N 117 | N 92 | N 23 | N 92 | N 27 | N 351 |  |
| Non hazardous use | 87 (74.4%) | 58 (63.0%) | 17 (73.9%) | 64 (69.6%) | 17 (63.0%) | 243 (69.2%) | 0.43^1^ |
| Hazardous/harmful use | 30 (25.6%) | 34 (37.0%) | 6 (26.1%) | 28 (30.4%) | 10 (37.0%) | 108 (30.8%) |  |

^1^ Tests used: Pearson's x^2^

*Category 1 - Answered questionnaires at the start, completion, and follow-up

*Category 2 - Answered questionnaires at the start and completion

*Category 3 - Answered questionnaires at the start and follow-up

*Category 4 - Answered questionnaires at the start

*Category 5 - Answered questionnaires at the start but were not included in the study, as they received less than five sessions of psychological treatment.

**Table 2** Mean values at the start of the psychological treatment by category for patients with hazardous/harmful alcohol use.

| **Hazardous/harmful use** | |  |  |  |  |  |  |
| --- | --- | --- | --- | --- | --- | --- | --- |
|  | **Category 1*** | **Category 2*** | **Category 3*** | **Category 4*** | **Category 5*** | **Total** | **P-value^1^** |
|  | N 30 | N 34 | N 6 | N 28 | N 10 | N 108 |  |
|  | Mean (SD) | Mean (SD) | Mean (SD) | Mean (SD) | Mean (SD) |  |  |
| BAI | 27.7 (12.8) | 25.9 (11.7) | 30.7 (12.3) | 27.6 (13.0) | 29.9 (15.1) |  | 0.861 |
| Madrs-S | 24.0 (7.5) | 26.3 (7.9) | 28.3 (11.1) | 25.0 (9.2) | 29.5 (11.9) |  | 0.411 |
| SCL-90 | 152.5 (48.3) | 166.5 (47.9) | 189.8 (52.7) | 157 (50.2) | 161.1 (94.8) |  | 0.491 |
| Q-Les-Q | 37.0 (8.0) | 35.6 (8.9) | 33.5 (7.5) | 37.9 (8.6) | 38.1 (13.2) |  | 0.821 |

^1^Tests used: Kruskal-Wallis test.

*Category 1 - Answered questionnaires at the start, completion, and follow-up

*Category 2 - Answered questionnaires at the start and completion

*Category 3 - Answered questionnaires at the start and follow-up

*Category 4 - Answered questionnaires at the start

*Category 5 - Answered questionnaires at the start but were not included in the study as they did not receive five or more sessions of psychological treatment.

**Table** **3** *Proportion of hazardous and non-hazardous alcohol use.* Comparison of the proportion of patients with non-hazardous and hazardous/harmful alcohol use at the start, with the proportion of the same grouping, at completion and follow-up, also disaggregated by gender. Proportion at completion and follow-up based on alcohol use level at the start of psychological treatment.

|  | **Non-Har use**  **at the start** | **Haz/Harm use**  **at the start** |  |  |
| --- | --- | --- | --- | --- |
| **All patients** | **N (%)** | **N (%)** | **N Tot** | **P-value^1^** |
| Start | 226 (69.8%) | 98 (30.2%) | 324 |  |
| Completion | 145 (69.4%) | 64 (30.6%) | 209 | 0.940 |
| Follow-up | 104 (74.3%) | 36 (25.7%) | 140 | 0.270 |
|  |  |  |  |  |
| **Female** |  |  |  |  |
| Start | 164 (72.6%) | 62 (27.4%) | 226 |  |
| Completion | 112 (72.3%) | 43 (27.7%) | 155 | 0.928 |
| Follow-up | 76 (72.4%) | 29 (27.6%) | 105 | 1.000 |
|  |  |  |  |  |
| **Male** |  |  |  |  |
| Start | 62 (63.3%) | 36 (36.7%) | 98 |  |
| Completion | 33 (61.1%) | 21 (38.9%) | 54 | 0.778 |
| Follow-up | 28 (80%) | 7 (20%) | 35 | 0.052 |

^1^ Tests used: Binomial test - test of difference in proportion

**Table** **4** Response rate to outcome measures, based on the response category to which patients belonged at the start of their psychological treatment. Of the 20 patients with abuse or dependence on alcohol, 12 were women and 8 were men.

|  | | | |  |
| --- | --- | --- | --- | --- |
|  | **Start** | **Completion** | **Follow-up** | |
| Non hazardous use | 226 | 145 | 104 | |
| Hazardous use | 78 | 52 | 28 | |
| Alcohol abuse | 15 | 12 | 8 | |
| Dependence | 5 | 0 | 0 | |
| **Total** | 324 | 209 | 140 | |
